# Anasarca as a manifestation of childhood volvulus: Diagnostic and management challenges in an 11-year old Nigerian boy

**DOI:** 10.22088/cjim.14.1.138

**Published:** 2023

**Authors:** Adefunke Olarinre Babatola, Joshua Taye Ige, Adewuyi Temidayo Adeniyi, Ibukun Anuoluwa Abidoye, Olarewaju Joseph, Joshua Olorunfunmi, Benjamin Folorunsho Ilori, Akinwumi Kolawole Komolafe, Oladele Simeon Olatunya

**Affiliations:** 1 Department of Pediatrics and Child Health, Ekiti State University Teaching Hospital, Ado Ekiti, Ekiti State, Nigeria; 2 Department of Surgery, Ekiti State University Teaching Hospital, Ado Ekiti, Ekiti State, Nigeria; 3 Department of Radiology, Afe Babalola University/Multisystem Hospital, Ado Ekiti, Ekiti State, Nigeria

**Keywords:** Anasarca, Small intestine volvulus, Nigerian boy

## Abstract

**Background:**

Small intestine volvulus occurs more commonly among younger children. It often poses diagnostic challenges when it occurs in older children and adults. For good clinical outcomes, it is essential to have prompt presentation, diagnosis and early intervention. Anasarca is not a common clinical manifestation of small intestine volvulus.

**Case Presentation:**

We report this unusual presentation of small intestine volvulus in an 11-year old Nigerian boy who first presented only with anasarca. While being investigated for the cause of the anasarca, he developed features of acute abdomen thought to be spontaneous bacteria peritonitis initially. He had surgery where the diagnosis of small intestine volvulus was made.

**Conclusion:**

The diagnosis and management of both anasarca and small intestine volvulus could be fraught with challenges. It is possible that anasarca can be the first manifestation of small intestine volvulus.

Intestinal volvulus is a surgical emergency. It occurs when a loop of intestine twists around itself and the mesentery that supplies it, causing a bowel obstruction (1). It can affect the small and the large intestine. Small intestine volvulus is more common in children and it is often associated with congenital mal-rotation and therefore commonly present in the early childhood years particularly in the newborn period (2–4). Documented predisposing factors include duplication cysts, meconium ileus, jejunal atresia and stenosis, tumors, or Meckel diverticula (2,4). Small intestine volvulus is classified into two forms; primary small intestine volvulus where there is no apparent cause/predisposing anatomic defect and secondary small intestine volvulus when it is associated with predisposing anatomic defects. Small intestine volvulus often presents with bilious vomiting and abdominal pain in newborn, its presentation is however variable in older children; abdominal pain, abdominal distension, vomiting, constipation, and passage of bloody stools are common presentations (1). When it is not detected and treated early, it is often followed by life threatening conditions such bowel ischemia, necrosis and perforation. Abdominal ultrasound, upper gastrointestinal tract series and computed tomography are useful investigations to make diagnosis of small intestine volvulus, though none is accurate. Treatment is by surgical intervention. Anasarca is defined as massive and generalized edema. It is often a symptom and its causes include organ failure (heart, liver, kidney), liver cirrhosis, pregnancy, venous obstruction, lymphatic obstruction, burns, trauma and malignancy particularly in the adult population (5). However, the most common causes of anasarca in childhood include nephrotic syndrome, protein losing enteropathy, protein energy malnutrition and allergic reactions (6-8). 

The pathophysiology of anasarca often involves three major mechanisms which are reduced oncotic pressure, increased hydrostatic pressure and increased capillary permeability and occasionally lymphatic obstruction (5). History, examination and investigations targeted at some of the causes of anasarca are often employed by clinicians to unravel diagnosis in patients who present with anasarca. The management of anasarca is mostly dependent on its cause (5). Because of the array of causes of anasarca, sometimes, it may be quite challenging to make a diagnosis. We report this unusual presentation of small intestine volvulus as anasarca in an 11-year-old Nigerian boy.

## Case Presentation

An 11-year-old boy presented at our Children Emergency Ward (CEW) with generalized body swelling noticed one week prior to presentation. The swelling was first noticed at the feet, progressed cranially to involve the whole body (face, abdomen, scrotum and lower limbs). There was no reduction in urine output, no frothiness of urine and urine color was amber. Patient had no cough, no difficulty with breathing, no easy fatigability, no headache, no fever, no yellowness of the eyes, no itching and no passage of pale bulky stool. On examination, he had generalized body swelling, was not in obvious respiratory distress, not pale, anicteric, not cyanosed, not febrile, no palpable peripheral lymph nodes enlargement and no digital clubbing. He had bilateral pitting pedal edema and scrotal edema. He had grossly distended abdomen, no tenderness and no palpable organ enlargement. Ascites was present.

A provisional diagnosis of Anasarca Cause was made. Complete blood count and serum electrolytes, urea and creatinine (E/U/Cr) tested normal. Urinalysis showed 1+ proteinuria only, liver function test (LFT) showed reduced total protein of 35.5g/L (normal values-58-80g/L) and albumin of 20.2g/L (normal values- 35-50g/L). Other parameters of the LFT were normal. He had abdominal ultrasound which revealed ascites only. Stool examination for ova, parasite, microscopy and occult blood was normal. Stool for biochemical studies could not be done because of unavailability of facility. Chest x-ray was normal.

He was admitted, transfused with fresh frozen plasma and also had diuretics. He improved as the edema regressed significantly. He was discharged after 48 hours of admission to complete investigations on outpatient basis.

**Figure 1:**
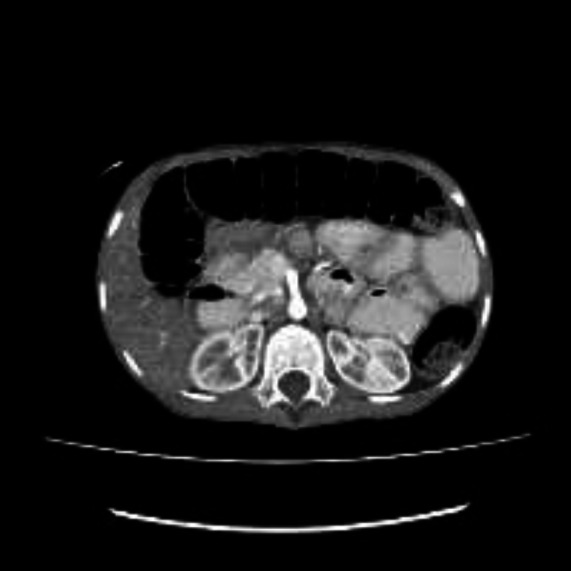
Axial CT at the levels of the kidneys, showing dilated small bowel loops (blue arrows)

**Figure 2:**
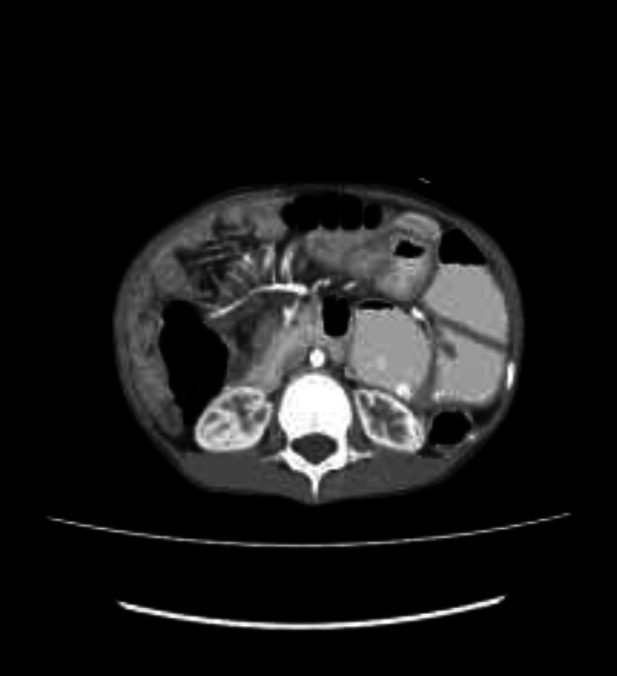
Showing hyper vascular appearance of the mesentery; comb sign (white arrow)

**Figure 3:**
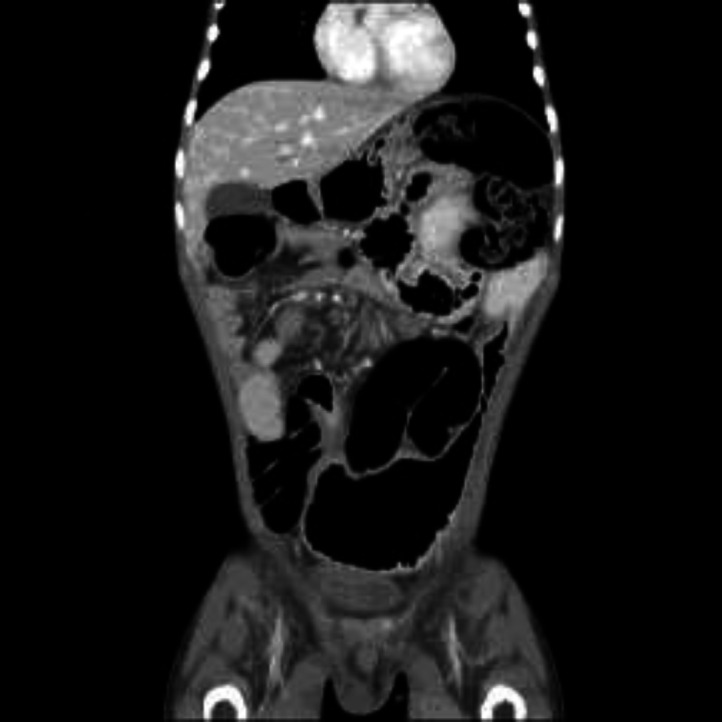
Coronal reformatted CT image showing alternate thickening of the wall of the terminal ileum (white arrow) and adjacent normal wall thickness (blue arrow), suggesting skip lesions in the terminal ileum

Three weeks later, he represented to the clinic with recurrent vomiting and occasional abdominal pain and recurrence of the generalized body swelling of 2 weeks duration. He was reported to have started vomiting about 2 weeks prior to this presentation, had about 2-3 episodes per day mostly postprandial, not bilious, not copious and content has recently ingested feeds. The vomiting was accompanied by occasional central abdominal pain. There was no constipation, no passage of watery stool and no fever. Patient was asked to do abdominal computed tomography scan. Two days later the report of the CT scan was brought to the clinic which showed marked dilatation of the small bowel loops up to the ileum. Multiple areas of thickening of the walls of the ileum with luminal narrowing interspersed by relatively preserved segments (skip lesions) were seen. No small bowel mass, stricture or fistula. The mesentery also appeared hyper vascular giving the comb sign (figures 1-3). The conclusion of CT scan findings was suggestive of Crohn’s disease with small bowel obstruction. At the clinic, he also complained of sudden abdominal pain of two hours duration. He was transferred to the CEW where physical examination showed he was in severe painful distress, febrile, severely dehydrated in shock and had generalized edema. The abdomen was grossly distended, moves minimally with respiration, generalized tenderness, bowel sounds was hypoactive. Diagnosis of spontaneous bacteria peritonitis was considered. He was managed with intravenous fluids, intravenous antibiotics and the pain reduced significantly. He had a repeat LFT which showed only reduced total protein of 36.9g/L (normal values-58-80g/L) and albumin of 20.2g/L (normal values- 35-50g/L). Serum E/U/Cr was normal. 

**Figure 4:**
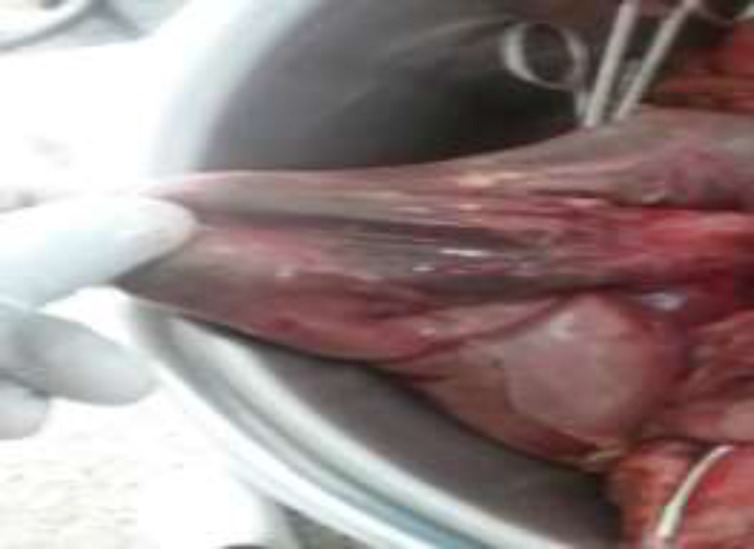
Gangrenous segment of the small intestine as shown by the blue arrows

**Figure 5:**
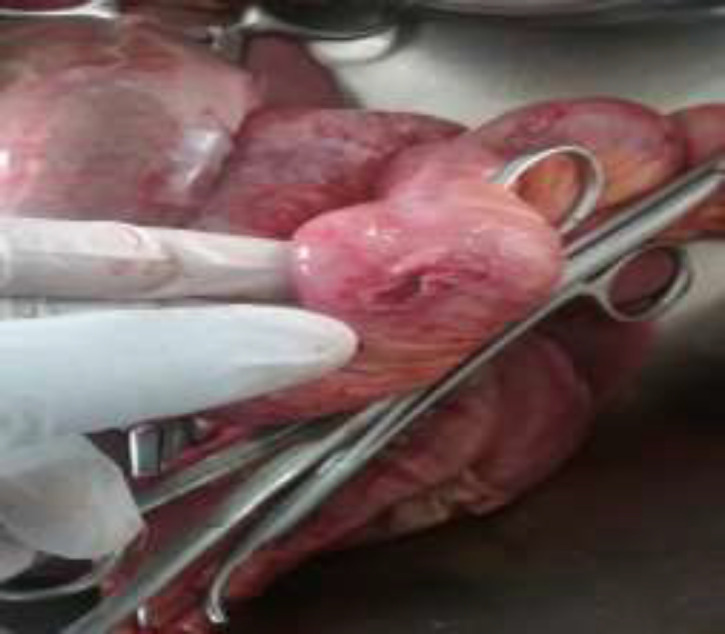
Perforation of a segment of the small intestine as shown by the blue arrow

On the 2^nd^ day of admission, he had 3 episodes of bilious vomiting and passage of bloody stools. Plain abdominal x-ray showed multiple air fluid levels and markedly dilated loops of bowel. The surgeons were invited and an impression of generalized peritonitis probably secondary to perforated viscus was made. The child had surgery and a diagnosis of small bowel volvulus was made intraoperatively. The other intra-operative findings were 2L of hemorrhagic peritoneal effluent, volvulus of 2.5 complete turns and gangrenous small bowel involved in the volvulus. He had bowel resection from the duodeno-jejunal junction to about 130cm before the recto-sigmoid junction with end-to end anastomosis (figures 4,5). Histopathology report of the resected bowel segment confirmed pan-mural necrosis with no evidence of malignancy.

He was stable post-operatively and was discharged 9 days after the surgery. One month after discharge, he was brought to the children emergency room with complaints of frequent passage of loose stools which contains undigested foods and often copious. On examination, he was cachectic, not in respiratory distress, not pale, afebrile, moderately dehydrated, anicteric and had pitting edema up to upper one-third of the legs. A diagnosis of acute diarrhea due to malabsorption from suspected short bowel syndrome with moderate dehydration was made.

He was re-admitted. Investigations revealed hypokalemia (1.9mmol/L), moderate anemia (25%), hypoproteinemia (40.1g/L) and hypoalbuminemia (19.1g/L). He was managed with intravenous fluids, antidiarrheal agent (loperamide) and also had blood transfusion. However, he died within 6 hours of admission of this readmission probably from electrolytes imbalances. Post-mortem examination was declined by parents.

## Discussion

History, clinical examination and investigations are often targeted at known common causes of generalized edema (nephrotic syndrome, acute glomerulonephritis, kwashiorkor, congestive cardiac failure, liver problems) when a child present with anasarca (9-11). Other less common documented causes of anasarca in childhood that are considered include protein losing enteropathy, severe burns, angioneurotic oedema, anteriovenous fistula, Kawasaki disease, Systemic lupus erythematosus and idiopathic (9-12). Patients with these clinical conditions usually have in addition to edema some features which point to them.

Small intestine volvulus is mostly a disease of infants and younger children. (13). Its diagnosis often poses major challenges when it occurs in older children and adults (14). The clinical features of small intestine volvulus are variable in older children and adults (15,16) and include abdominal pain, vomiting, constipation, with abdominal distension (16).

This index patient, an 11-year-old boy presented with anasarca only at his first presentation. The clinicians suspected nephrotic syndrome, though the progression of the edema was not in support, there was no passage of frothy urine, urinalysis revealed only 1+ of proteinuria as against 3+ or more which suggests nephrotic syndrome. That the urinalysis of our patient revealed 1+ proteinuria can be related to “physiologic proteinuria” where healthy children can excrete minimal protein in urine or transient proteinuria which is associated with fever, exercise, stress or cold exposure in children (17-19). However, serum protein and albumin were reduced on all the 3 occasions, they were assayed for in our patient. The cause of hypoproteinemia and hypoalbuminemia was not obvious in our patient.

Though our patient presented with recurrent vomiting and abdominal pain in addition to the anasarca at his 2^nd^ presentation at our facility, the presence of edema was confusing. There is no single investigation that is documented to be able to diagnose small intestine volvulus accurately (14). Abdominal imaging procedures were believed to be helpful (14,16). CT scan diagnosed small intestine volvulus with 45% accuracy (16) and this was confirmed in this case as our patient’s CT scan report was that of small intestinal obstruction with Crohn’s disease rather than volvulus. Hence, the clinicians thought that Crohn’s disease which has been documented to be associated with protein losing enteropathy will be able to explain the edema, (20) however, there were no other supporting features of Crohn’s disease (bloody diarrhea, weight loss, anemia) (21). The sudden worsening of abdominal pain and fever which necessitated presentation and admission at CEW was suspected to be spontaneous bacteria peritonitis because of the persistent anasarca and he was managed for this initially. The patient later passed bloody stool on admission, was reviewed by surgeons and had surgery where the diagnosis of small intestine volvulus with perforation and gangrene was made. That the diagnosis of small intestinal volvulus was made at surgery have been reported by previous authors (15, 22-24). This highlights the challenges with preoperative diagnosis of small intestine volvulus. Our case was also complicated by the presence of anasarca which contributed to the delay in making the diagnosis of small intestine volvulus. 

Our patient’s intraoperative findings were suggestive of complicated small intestine volvulus and may be related to late intervention resulting from diagnostic challenges. Unfortunately, our patient died about 6 weeks after surgery from features suggestive of short bowel syndrome and electrolyte imbalances which are known complications of volvulus surgery due to extensive bowel resection (25,26). 

 This case report highlights the diagnostic challenges that may be associated with anasarca and small intestine volvulus. It also reminds clinicians the need to consider small intestine volvulus in any child presenting with recurrent abdominal pain to prevent late intervention and its attendant problems. Furthermore, it calls for clinicians to have a high index of suspicion regarding making the diagnosis of volvulus in children. 

### Patient consent:

Written informed consent for participation and publication of medical details was obtained from the parents of the child. 
